# Ihh and Runx2/Runx3 Signaling Interact to Coordinate Early Chondrogenesis: A Mouse Model

**DOI:** 10.1371/journal.pone.0055296

**Published:** 2013-02-01

**Authors:** Eun-Jung Kim, Sung-Won Cho, Jeong-Oh Shin, Min-Jung Lee, Kye-Seong Kim, Han-Sung Jung

**Affiliations:** 1 Division in Anatomy and Developmental Biology, Department of Oral Biology, Research Center for Orofacial Hard Tissue Regeneration, Brain Korea 21 Project, Oral Science Research Center, College of Dentistry, Yonsei University, Seoul, Korea; 2 Graduate School of Biomedical Science and Engineering, College of Anatomy and Cell Biology, Hanyang University, Seoul, Korea; University of Massachusetts, United States of America

## Abstract

Endochondral bone formation begins with the development of a cartilage intermediate that is subsequently replaced by calcified bone. The mechanisms occurring during early chondrogenesis that control both mesenchymal cell differentiation into chondrocytes and cell proliferation are not clearly understood in vertebrates. Indian hedgehog (Ihh), one of the hedgehog signaling molecules, is known to control both the hypertrophy of chondrocytes and bone replacement; these processes are particularly important in postnatal endochondral bone formation rather than in early chondrogenesis. In this study, we utilized the maternal transfer of 5E1 to E12.5 in mouse embryos, a process that leads to an attenuation of Ihh activity. As a result, mouse limb bud chondrogenesis was inhibited, and an exogenous recombinant IHH protein enhanced the proliferation and differentiation of mesenchymal cells. Analysis of the genetic relationships in the limb buds suggested a more extensive role for Ihh and Runx genes in early chondrogenesis. The transfer of 5E1 decreased the expression of *Runx2* and *Runx3*, whereas an exogenous recombinant IHH protein increased *Runx2* and *Runx3* expression. Moreover, a transcription factor Gli1 in hedgehog pathway enhances the direct induction of both Runx2 and Runx3 transcription. These findings suggested that Ihh signaling plays an important role in chondrocyte proliferation and differentiation via interactions with Runx2 and Runx3.

## Introduction

Chondrogenesis, the process by which cartilage is formed, occurs following two processes: mesenchymal cell condensation and differentiation. After undifferentiated mesenchymal cells migrate to condensation [1], the prechondrocytes located in the center of the condensation differentiate into chondrocytes. Chondrocytes in the center of cartilage actively and continuously undergo differentiation processes such as proliferation and maturation [2], [3]. The developing limb of vertebrates is as an excellent model system to study patterning [4] and endochondral bone formation [3]. Endochondral bone formation in limbs, where extensive growth is required for the proximal–distal extension of the long bones, also begins with the aggregation and condensation of undifferentiated mesenchymal cells whose position, shape, and size dictate the morphology of future skeletal elements.

A large number of growth factors, such as Bmps (Bone morphogenetic proteins), Fgfs (Fibroblast growth factors), Wnts, Ihh (Indian hedgehog), and PTHrP (Parathyroid hormone-related protein), are expressed in chondrocytes. *Ihh* is first expressed at embryonic day (E) 12.0 by the chondrocytes at the center of condensation in the cartilage of the mouse limb bud [5] and is later expressed in periarticular cells and in articular chondrocytes at sites of prehypertrophic differentiation by the formation of a negative feedback loop. Ihh activates the expression of *PTHrP*, PTHrP signals inhibit chondrocyte hypertrophy, and further suppress *Ihh* expression by maintaining chondrocytes in a proliferating state [6], [7], [8]. Ihh signaling is also required for chondrocyte proliferation and osteoblast differentiation, independent of PTHrP signaling [9], [10].

Most of the *Ihh* ^−/−^ mice die at birth due to respiratory failure resulting from defects in the development of the rib cage [8]. The first overt sign of an embryonic phenotype in these mice is an obvious shortening of the forelimbs at E13.5, and these mice are invariably shorter in length at birth than wild-type mice. The proliferation and hypertrophy of chondrocytes are significantly reduced in *Ihh* ^−/−^ mice, indicating that these chondrocytes are the likely targets of Ihh signaling. The disorganization of the mesenchymal cells surrounding the cartilage condensation at E12.5 and the thinner perichondrium at E14.0 in *Ihh* ^−/−^ mice suggest that Ihh is required for the proper segregation of cells into a perichondrial lineage and for subsequent osteoblast differentiation [11].

Runx2 (Runt-related transcription factor 2) is a transcriptional factor of the specific genes involved in chondrocyte hypertrophy and osteoblast differentiation [12], [13]. *Runx2* is highly expressed in the developing limb cartilage of mice beginning at E12.5 [14]. *Runx2* expression in the perichondrium of mouse embryos was found to be dependent on the expression of *Ihh* in chondrocytes [8], and the absence of *Runx2* expression in the perichondrium/periosteum of *Ihh* ^−/−^ mice was reported beginning at E14.5 [15]. Furthermore, treatment with recombinant SHH (Sonic hedgehog) protein in chondrocytes elevated the expression of *Runx2* and enhanced the activity of the 1.8 kb Runx2 promoter. Therefore, it was suggested that Runx2 is regulated by the hedgehog protein in chondrogenesis [16]. Vice versa, it has been also reported that Runx2 directly regulates *Ihh* expression in chondrocytes [17].

Interestingly, a lack of Runx2 function significantly delays or eliminates the hypertrophy of chondrocytes in stylopods, such as the humerus and femur, rather than in other skeletal elements [17], whereas lacking the functions of both *Runx2* and *Runx3* blocks hypertrophy in the more distal skeletal elements. Runx3 has been known to cooperate with Runx2 in the regulation of chondrocyte differentiation and maturation [16].

The role of Ihh and its relationship with other genes in the regulation of chondrocyte proliferation and differentiation during early chondrogenesis has not yet been fully understood. Here, the maternal transfer of 5E1 (an IgG1 monoclonal antibody against the hedgehog protein) through the placenta was utilized to block hedgehog signaling in mouse embryos [18], [19]. The changes in the phenotype and the gene expression patterns of the mouse limbs were investigated. Additionally, the changes in expression of the downstream target molecules in the hedgehog signaling pathway that occur during the early stages of chondrogenesis, particularly Runx2 and Runx3, were investigated. This study demonstrates how the inhibition of Ihh activity, which regulates Runx2 and Runx3 in the perichondrium during early chondrogenesis, results in limb shortening.

## Materials and Methods

All experiments were approved by and performed according to the guidelines of the Yonsei University, College of Dentistry, Intramural Animal Use and Care Committee.

### Drug Delivery

The monoclonal antibody (mAb) 5E1, an IgG1 monoclonal antibody against the Hedgehog protein, was obtained from hybridoma cells at the Developmental Studies Hybridoma Bank, developed under the auspices of the National Institute of Child Health and Human Development and maintained by the University of Iowa, Department of Biological Sciences (Iowa City, IA, USA). A single injection of 5E1 (20 mg/kg body weight) or phosphate-buffered saline (PBS, 1 ml) was administered intraperitoneally to pregnant ICR mice (purchased from Koatech Co, Pyeongtaek, Korea) on E12.5 (n = 5), E14.5 (n = 5), E16.5 (n = 5), and E18.5 (n = 5).

The newborn mice were allowed lifespans of one week, three weeks, or six weeks, and after these times, they were sacrificed, stained with alizarin red and alcian blue and subjected to skeletal analysis.

### MicroCT

Three-dimensional reconstructed computed tomography images were obtained by scanning calcified bone using micro-computed tomography (PBS, n = 5; 5E1, n = 5) (Micro-CT, Skyscan 1076, Skyscan, Antwerp, Belgium). The data were then digitalized using a frame grabber, and the resulting images were transmitted to a computer for analysis using Comprehensive TeX Archive Network (CTAN) topographic reconstruction software. The lengths of the humerus, ulna, and radius were measured from the head to the medial epicondyle, from the olecranon to the styloid process, and from the head to the styloid process, respectively.

### Microarrays

Gene expression was measured using a mouse gene microarray (Gene Chip Mouse Genome 430 2.0, Affymetrix, Santa Clara, CA, USA) with RNA from the limb buds of the embryos of pregnant mice at one day after injection (PBS, n = 5; 5E1, n = 5). A gene-chip scanner (Gene Chip Scanner 3000, Affymetrix) was used to measure the intensity of the fluorescence emitted by the labeled target. Raw image data were converted to cell-intensity (CEL) files using the Affymetrix Gene Chip Operating System, and the resultant CEL files were normalized using the MAS 5.0 algorithm. Following statistical analysis, differentially expressed genes were selected using GenePlex software version 3.0 (ISTECH, Korea). Differentially expressed genes in Wnt, TGF- β, Hedgehog, and Fgf signaling pathways with changes in expression of at least 1.5-fold in the 5E1-treated group compared to the control group were selected. These genes were analyzed using Student’s t-test, with the level of statistical significance set at *p*<0.01.

### Quantitative Reverse-transcriptase Polymerase Chain Reaction (RT-qPCR)

RNA was extracted from the forelimbs of embryos one day after injection (PBS, n = 5; 5E1, n = 5). RT-qPCR was performed using a Thermal Cycler Dice Real-Time System and SYBR Premix EX Taq (Takara, Japan) according to the manufacturer’s instructions. The reaction for RT-qPCR was initially incubated for one min at 95°C, and the amplification program consisted of 40 cycles of the following steps: denaturation at 95°C for 5 sec, annealing at 55–60°C for 10 sec, and extension at 72°C for 10 sec. RT-qPCR was performed in triplicate for each sample, and the amounts of each of the RT-qPCR products were normalized using β-2-microglobulin as an internal control. The primers used for amplification were as follows: B2m (beta-2 microglobulin), 5′-GGGAAGCCGAACATACTGAA-3′ and 5′-TCACATGTCTCGATCCCAGT-3′; Bmp4 (bone morphogenetic protein 4), 5′-CCATCACGAAGAACATCTGG-3′ and 5′-TGTGATGAGGTGTCCAGGAA-3′; Bmp5 (bone morphogenetic protein 5), 5′-AACAAGCCTGCAAGAAGCAC-3′ and 5′-TACAGGACCGAGATGGCATT-3′; Gli1 (GLI-Kruppel family member GLI1), 5′-CTTCAAGGCCCAATACATGC-3′ and 5′-ATGGCTTCTCATTGGAGTGG-3′; Hhip (Hedgehog-interacting protein), 5′-ATGCCTGAGGAATGCAGAGT-3′ and 5′-CGTTCCTGTCCACTTGTTCA-3′; Ihh (Indian hedgehog), 5′-GGCTTCGACTGGGTGTATTA-3′ and 5′-CGGTCCAGGAAAATAAGCAC-3′; Ptch1 (patched homolog 1), 5′-AAGCCACAGAAAACCCTGTC-3′ and 5′-TATTGCTAGGGCCAGAATGC-3′; Runx2 (runt related transcription factor 2), 5′-AGATGACATCCCCATCCATC-3′ and 5′-GTGAGGGATGAAATGCTTGG-3′; Runx3 (runt related transcription factor 3), 5′-GCCGGCAATGATGAGAACTA-3′ and 5′-AGGCCTTGGTCTGGTCTTCTAT-3′; and Sox9 (SRY-box containing gene 9), 5′-CAAGAACAAGCCACACGTCA-3′ and 5′-TGTAATCGGGGTGGTCTTTC-3′. The data were analyzed using Thermal Cycler Dice Real-Time System software and the 2^−ΔΔCt^ method [20]. Statistical analysis was performed using Student’s t-tests of variables to determine significant changes at a 95% confidence level (*p*<0.05).

### Micromass Culture

The limb buds were dissected from E12.5 embryos in calcium−/magnesium-free saline (CMF), placed in 1.2 units/ml of dispase II (Roche, Mannheim, Germany) in CMF at 37°C for 25 min and washed, and the ectoderm was removed using a tungsten needle. Mesenchymal cells were gently dissociated in 1–2 ml of medium to produce a single-cell suspension. The cells were suspended at a density of 2×10^7^ cells/ml in 60% culture medium (Nutrient mixture F-12 Ham with 10% fetal calf serum, 1–4 mM-L-glutamine, 1% penicillin streptomycin and 200 µg/ml ascorbic acid with 40% CMF containing 10% newborn calf serum). A single 10 µl drop of this suspension was plated onto each 35 mm tissue culture dish, and the dishes were placed in a humidified incubator with an atmosphere of 5% CO_2_/95% air at a temperature of 37°C. The cells were incubated for 50 min, then, flooded with 1–2 ml of culture medium. The cultures received fresh medium daily. For the hedgehog inhibition experiments, the cells were treated with 130 µg/ml of 5E1. The Ihh overexpression experiments were performed using 500 ng/ml of recombinant mouse IHH (R&D Systems, MN, USA). After 4 days, the number of mesenchymal aggregates was assessed, and the cultures were fixed in 4% paraformaldehyde (PFA) in CMF and stained with alcian blue to assess the extent of chondrogenesis.

The alcian blue incorporated into the cell matrix was extracted with 0.5 ml of 4 M guanidine HCl (pH 5.8) and quantified by measuring absorbance at an optical density of 570 nm. The statistical difference in alcian blue staining between the control and experimental micromass cultures was assessed using the nonparametric Wilcoxon signed rank test. A value of *p<0*.01 was considered a statistically significant difference.

### Protein Bead Implantation

Cell pellets composed of 1.5×10^6^ mesenchymal cells from the E12.5 limb buds were implanted with beads soaked in PBS or 1 mg/ml of IHH protein. Affi-Gel blue beads (Bio-Rad Laboratories, Hercules, CA, USA) were soaked with 1 mg/ml of the recombinant IHH protein at room temperature for one hour, and the control beads were soaked in PBS. The beads were placed with the E12.5 limb bud mesenchymal cell pellets, which were then cultured for three hours or 24 hours in DMEM plus 10% FBS. Whole-mount *in situ* hybridization and RT-qPCR were then performed.

### *In situ* Hybridization

Tissues were fixed overnight in 4% PFA. The hybridizations were performed on the limb buds with digoxigenin-labeled RNA probes in hybridization buffer for 18 hours at 72°C, and the hybridization signals were detected using alkaline phosphatase-conjugated, anti-digoxigenin antibodies with a nitro blue tetrazolium chloride/5-bromo-4-chloro-3-indolyl phosphate toluidine salt substrate (Roche, Mannheim, Germany). The Runx2 cDNA was kindly provided by professor Je-Yong Choi (Kyoungpook National University, Korea).

### Western Blot

Total protein was extracted from the 5E1- or PBS-injected limb buds, which were harvested two days after injection at E12.5. The limb buds were lysed via sonication device (Nextadvance) in RIPA buffer (50 nM of Tris pH 7.5, 150 mM NaCl, 1 mM EDTA, 1% Triton X-100) for Western blot analysis. The following antibodies were used: anti-α-tubulin (1∶1,000, Sigma, MO, USA), anti-Runx2 (1∶100, Novus, CO, USA) and anti-Runx3 (1∶100, Abcam, Cambridge, UK). Horseradish peroxidase conjugated secondary antibodies (1∶10,000, SantaCruz, CA, USA) were used and protein bands were visualized by enhanced chemiluminescence (Amersham Biosciences, Buckinghamshire, UK).

### Luciferase Assay

PCR was used to amplify 1 kb of the Runx2 promoter region, including the Gli-binding sites (TGGGTGTTC, AGGGTGGCT) from -1380 bp to -381 bp (primers used: sense 5′- ACACACTTACGAAGTAAGCGGAGAGCTCTT-3′ and anti-sense 5′- TTTTCACAATCGGACCCCTAAGGCCTAGGACA-3′). 692 bp of the Runx3 promoter region, from -670 bp to +22 bp (primers used: sense 5′-CCCCTCGAGCCAGTACCTCATGT-3′ and anti-sense 5′-CCACTCGAGAGACTCAGCAGCCTG-3′), including the Gli-binding site (TGCCACCCA) was amplified by PCR. These sequences were inserted into the pGL3 basic vector to construct Runx2-luciferase reporter (pGL3-Runx2), and Runx3-luciferase reporter (pGL3-Runx3). Mutation form in the one of Gli-binding site (TGCCAGTTC) in Runx2 was induced by using primers (sense 5′-CCTTCTGTCTCTTTACTTATGCCAGTTCCTCTGTCTCTCCTTCC-3′ and anti-sense 5′-GAAGGAGAGACAGAGGAACTGGCATAAGTAAGAGACAGAAGG-3′). Mutation form in the one of Gli-binding site (TGCCTGGCA) in Runx3 was induced by using primers (sense 5′-AAAACCCCCTGAAGTGCCTGCAGCCACATATGTGGATG-3′ and anti-sense 5′- CATCCACATATGTGGCTGCCAGGCACTTCAGGGGGTTTT-3′). 293T (human embryonic kidney cells) cells were seeded at a density 5×10^5^ cells/cm^2^. After 24 hours, Runx-containing reporter plasmid and the pFLAG-CMV3:Gli1 plasmid were cotransfected into 293T cells using the FuGENE HD transfection reagent (Roche, Mannheim, Germany). An empty pGL3-basic plasmid, pRL-TK, the Renilla luciferase vector, and pFLAG-CMV3:Gli1 were also cotransfected to standardize the transfection efficiency. Luciferase assays were performed 48 hours post-transfection using the dual-luciferase assay system (Promega, WI, USA). Luciferase activity was determined using specific substrates in a luminometer (Promega, WI, USA) according to the manufacturer’s protocol.

## Results

### Short Limbs are Induced by Blocking Ihh Activity

**Figure 1:**
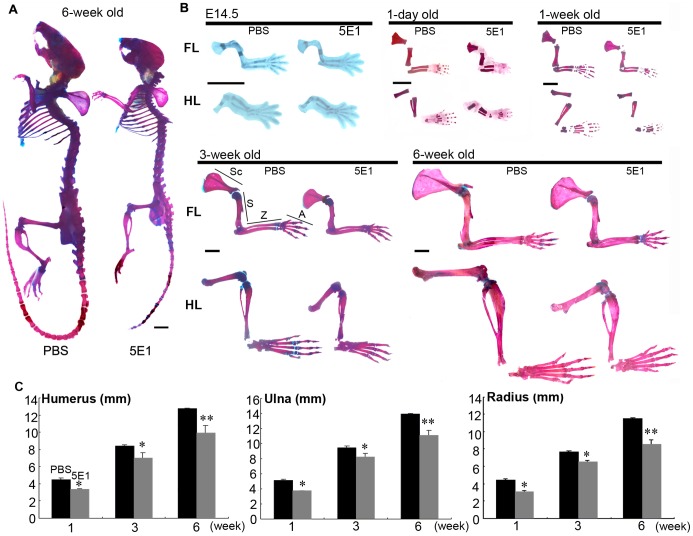
Skeletal staining with alcian blue and alizarin red and microCT of limb length. Skeletal staining was attempted with the PBS- and 5E1-injected mice. (A) 6-week old mice exposed to 5E1 at E12.5 exhibit limb, skull, tail and trunk bones that are reduced in size and length, but mice exposed to PBS show the same bone length as wild-type mice. (B) The forelimbs and hindlimbs of E14.5, one-day-old, one-week-old, three-week-old and six-week-old mice were stained with alcian blue and alizarin red. Mice injected with 5E1 have both shorter forelimbs and hindlimbs, and their bones grow similarly to those of PBS-injected mice. (C) The length of the humerus, ulna, and radius in 5E1-injected mice are shorter than in the PBS-injected mice at one-week old, three-week old, and 6-week old; the skeletal staining results agree with those of the microCT analysis. Student’s t-test was employed for statistical analysis, with the level of statistical significance set at (*) *p*<0.01 or (**) *p*<0.05 (Sc; Scapula, S; Stylopod, Z; Zeugopod, A; Autopod, scale bar = 3 mm).

6-week- old mice delivered from pregnant mice injected with 5E1 at E12.5 exhibited limb, skull, tail, and trunk bones that were reduced in size and length, whereas all mice delivered from pregnant mice injected with PBS as a control showed the same bone lengths and sizes as those of wild-type mice (Fig. 1 A). The forelimbs and hindlimbs of the 5E1-experimental group were significantly shorter in their proximal–distal length and smaller in size than those of the control group; this difference was also maintained in postnatal mice aged one day, one week, three weeks and six weeks. The lengths of cartilage and calcified bones in the stylopods, zeugopods and autopods of both the forelimbs and hindlimbs were shorter in the experimental group than in the control group (Fig. 1 B). The patterning of phalanges was not changed following the 5E1 injection because mice in the experimental group exhibited five phalanges. However, their phalangeal bones were shorter in length than those of the control group (Fig. 1 B). MicroCT confirmed that the humerus, ulna and radius in the forelimbs of the experimental group were shorter in length at one, three, and six weeks (Fig. 1 C). Mice treated with 5E1 at E14.5, E16.5 and E18.5 did not exhibit shorter limbs (data not shown), and no changes were observed in the injected pregnant mice. These results show that the lengths of cartilage and bone in limbs were reduced by the injection of 5E1 into pregnant mice at E12.5, when the cartilage primordium appears. These results suggest that Ihh signaling may be associated with the condensation of mesenchymal cells and their differentiation into chondrocytes to form the cartilage primordium.

### Maternal Transfer of 5E1 at E12.5 Effectively Blocks Ihh Activity

**Figure 2:**
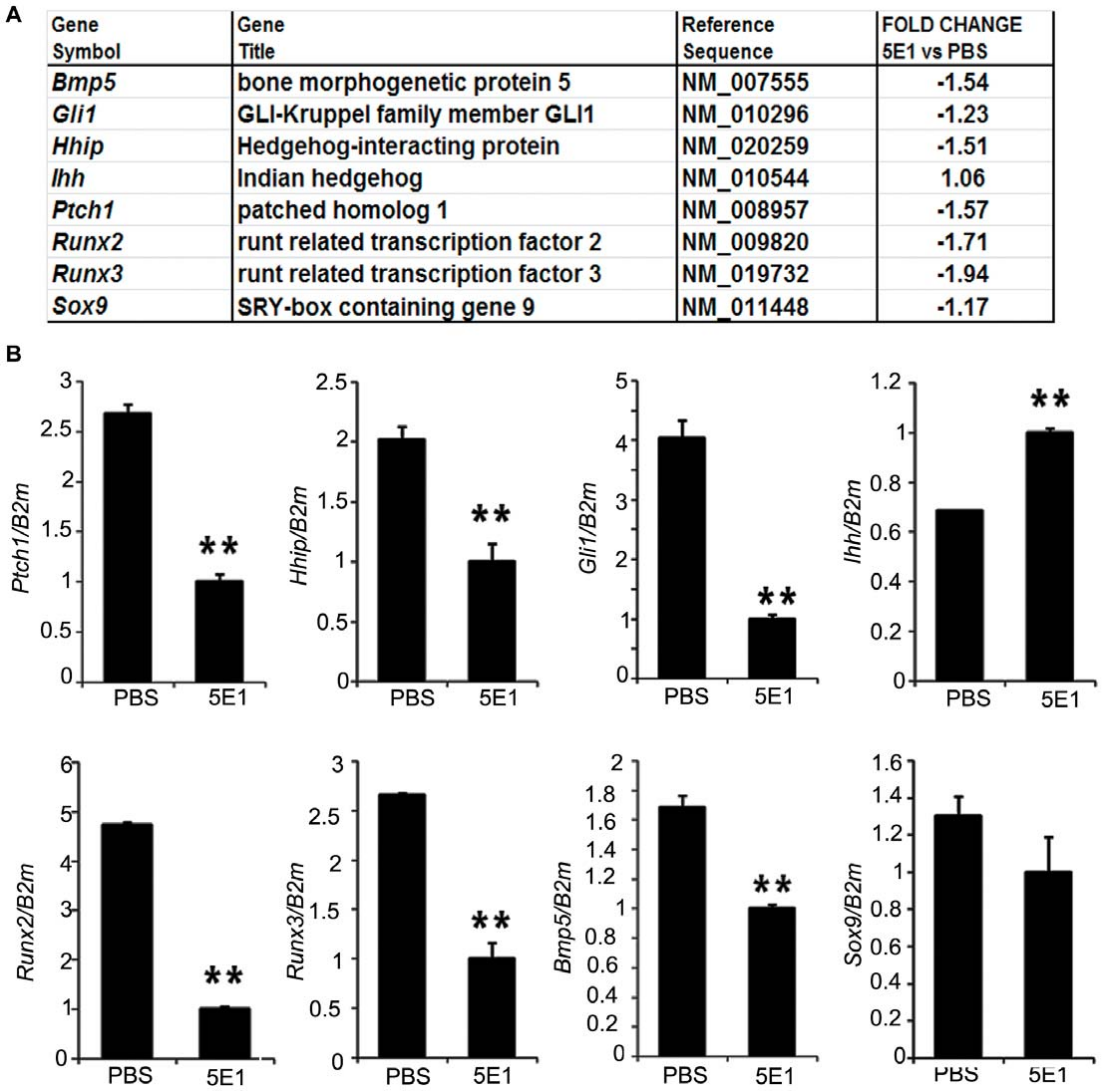
Microarray and RT-qPCR. (A) A table showing the fold change in the known chondrogenesis-related genes in limb between the groups injected with either PBS or 5E1 at E12.5. As expected, *Gli1, Hhip,* and *Ptch1* were down-regulated. Interestingly, *Runx2*, *Runx3, and Bmp5* were down-regulated. *Sox9*, which is the master gene in chondrogenesis, was not altered by 5E1 treatment. (B) The results of the RT-qPCR are consistent with the microarray data. The amount of each of the RT-qPCR products was normalized using β-2-microglobulin (B2m) as an internal control. Student’s t-test was used for statistical analysis with the level of statistical significance set at (*) *p*<0.01 or (**) *p*<0.05.

To examine the effects of 5E1 on Ihh signaling in developing limbs, the transcriptional profiles of limb buds were investigated using microarrays and RT-qPCR (Fig. 2 and Fig. S1). For the microarrays, embryos exposed to 5E1 or PBS were harvested one day after injection at E12.5. Both microarrays and RT-qPCR showed that *Ptch1* and *Hhip*, direct targets of hedgehog signaling, were significantly down-regulated in 5E1-treated limb buds (Fig. 2). Meanwhile, *Gli1*, another target of hedgehog signaling, was slightly down-regulated, as shown by microarrays, but was significantly down-regulated, as shown by RT-qPCR. In contrast, the expression levels of *Gli2*, *Gli3* and *Smo*, which are not activated in response to hedgehog signaling but are involved in the transcription of hedgehog signal genes [21], were not changed following 5E1 injection (Fig. S1). Furthermore, the *Ihh* expression level was increased, as shown by RT-qPCR (Fig. 2 B).

**Figure 3:**
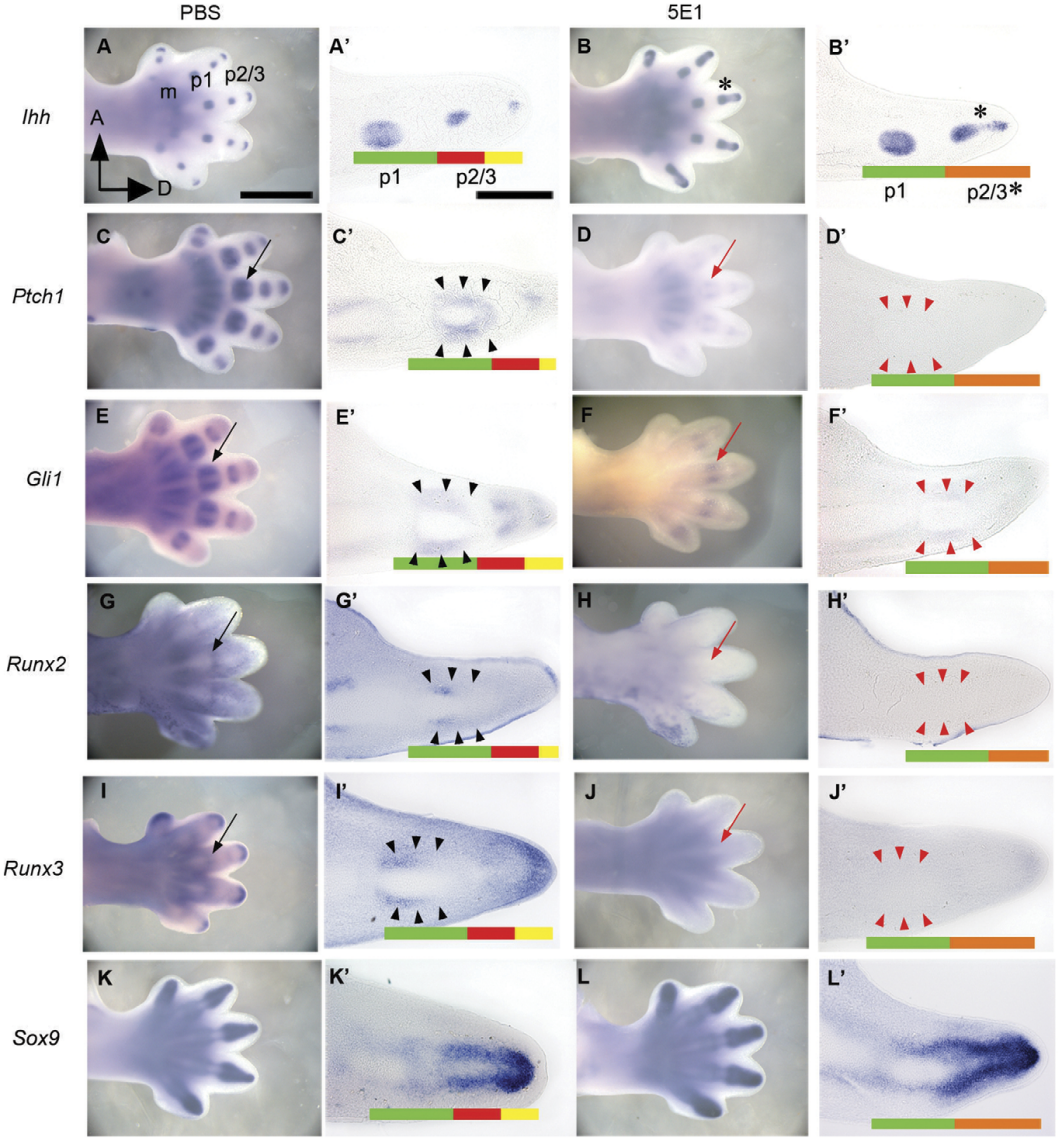
Expression of *Ihh*, *Ptch1*, *Gli1, Runx2, Runx3* and *Sox9.* (A and A’) Ihh expressed in prehypertrophic chondrocytes. (B and B’) The *Ihh* expression pattern was not altered when hedgehog signaling one day of 5E1 injection at E12.5. The *Ihh*-expressing regions in second and third phalanges were connected following the injection of 5E1, but not after injecting PBS. (C and C’, E and E’) *Ptch1* and *Gli1* were expressed in the mesenchymal cells surrounding the prehypertrophic chondrocytes. (D and D’, F and F’) *Ptch1* and *Gli1* were down-regulated due to blocking the activity of the IHH protein. (G and G’, I and I’) *Runx2* and *Runx3* were expressed in mesenchymal cells, similar to *Ptch1* and *Gli1*. (H and H’, J and J’) *Runx2* and *Runx3* were down-regulated when 5E1 was transferred. (K–L’) *Sox9* expression did not differ between the PBS- and 5E1-treated groups. p1 (green box), condensation of phalange 1; p2/3 (red and yellow box), unseparated primordium of phalanges 2 and 3; p2/3* (orange box), connecting region of Ihh expression; m, metacarpals; A, anterior; D, distal part of the limb; (A–L, scale bar = 1 mm. A’–L’; longitudinal section view, scale bar = 100 µm.).

Moreover, changes in gene expression profiles were investigated using *in situ* hybridization of members of the hedgehog family and downstream genes, such as *Ptch1* and *Gli1*, one day after blocking Ihh at E12.5. Because the 5E1 antibody binds to the IHH protein, *Ihh* mRNA expression was still observed in the experimental group (Fig. 3 A and A’, B and B’). *Ihh* was expressed in the prehypertrophic chondrocytes of developing cartilage in both the experimental (Fig. 3 A and A’) and control limbs (Fig. 3 B and B’). Interestingly, the *Ihh*-expressing regions in the primordia of phalanges two and three were connected (Fig. 3 B and B’), contrasting with what was observed in the controls (Fig. 3 B and B’). *Ptch1* (Fig. 3 C and C’) and *Gli1* (Fig. 3 E and E’) were expressed in the mesenchymal cells surrounding the prehypertrophic chondrocytes that express *Ihh* in the control limb buds, whereas *Ptch1* (Fig. 3 D and D’) and *Gli1* (Fig. 3 F and F’) were not expressed in developing limbs in the experimental group.

### Multiple Genes are Down-regulated after Blocking Ihh Activity at E12.5

The expression levels of *Runx2* and *Runx3* were down-regulated almost two-fold after blocking Ihh signaling activity (Fig. 2 E and F). The expression patterns in the control group of *Runx2* (Fig. 3 G and G’) and *Runx3* (Fig. 3 I and I’) were similar to those of *Ptch1* (Fig. 3 C and C’) and *Gli1* (Fig. 3 E and E’). *Runx2* and *Runx3* were expressed in the putative perichondrial cells that surround the prehypertrophic chondrocytes expressing *Ihh*, whereas the expression of *Runx2* and *Runx3* disappeared in the limb buds of the experimental group (Fig. 3 H and H’, J and J’). Furthermore, the translational level of Runx2 and Runx3 were decreased in the experimental group (Fig. S2). These results suggest a close relationship between Runx2, Runx3 and the Ihh signaling pathway in early chondrogenesis.

The expression level (Fig. 2 H) or pattern (Fig. 3 K–L’) of *Sox9*, a key regulator of early chondrogenesis [22], was not significantly altered by the 5E1 injection. *Sox9* was strongly expressed in the condensing mesenchyme in phalanges two and three as well as in the perichondral region of phalange one in both the control and experimental groups (Fig. 3 K–L’). This result indicates that Sox9 may not be a downstream gene in the Ihh signaling pathway.

*Bmp5* was also found to be down-regulated in the microarrays (Fig. S1) and RT-qPCR (Fig. 2 G). *Bmp5* is also expressed in the region of the perichondrium that flanks the *Ihh* expression domain and promotes chondrocyte proliferation and hypertrophy, independent of PTHrP signaling [23]–[27]. A slight increase in Bmp signaling would result in slightly larger bones, whereas a slight decrease in Bmp signaling would result in shorter bones. Nevertheless, more research is needed to show whether the decreased expression of *Bmp5* reduced the length of limbs.

### Ihh Induces the Chondrogenesis of Limb Bud Mesenchymal Cells

**Figure 4:**
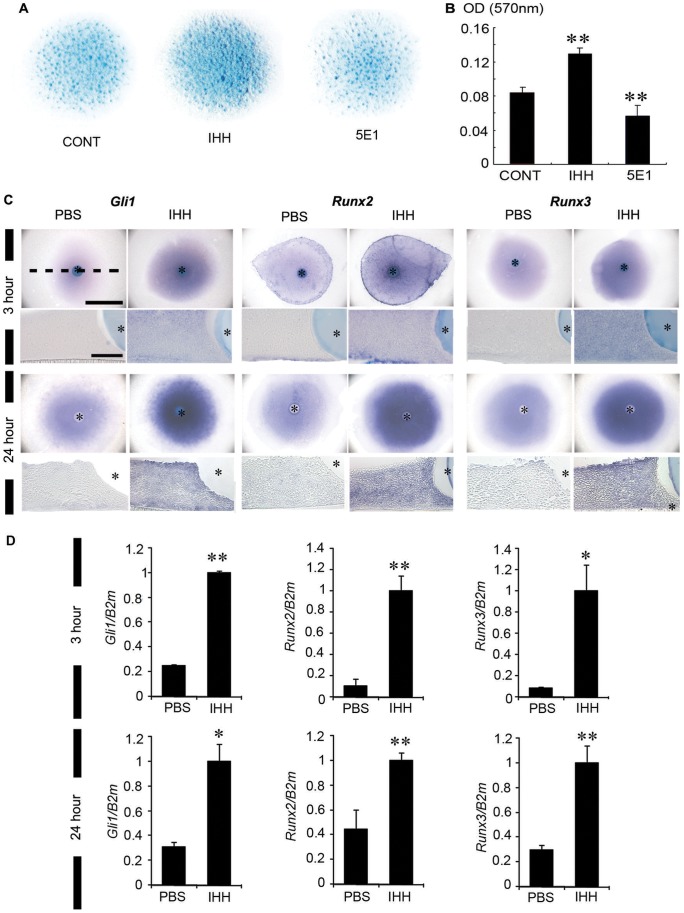
The exogenous IHH protein induced proliferation and differentiation of E12.5 limb bud mesenchymal cells and ectopic expression of *Runx2* and *Runx3*. (A, B) Cells were cultured at a density of 2×10^7^ cells/ml with 500 ng/ml of the IHH protein and 130 µg/ml of 5E1. The addition of exogenous IHH protein leads to the production of more cartilage nodules, whereas 5E1 treatment leads to a slight decrease in the formation of cartilage nodules. The value was quantified by measuring the absorbance of the bound alcian blue at 570 nm. (C) After the beads soaked in 1 mg/ml of IHH were implanted into 1.5×10^6^ mesenchymal cell pellets of limb buds at E12.5, followed by incubation for 3 or 24 hours, ectopic *Gli1*, *Runx2* and *Runx3* expression appear strongly around the IHH protein-soaked beads. (D) The *Gli1*, *Runx2* and *Runx3* expression levels are up-regulated by the exogenous IHH protein. Student’s t-test was employed for statistical analysis, with the level of statistical significance set at (*) *p*<0.01 or (**) *p*<0.005 (scale bar = 1 mm in the whole view, and scale bar = 100 µm in the section view of the cell pellets).

To determine whether Ihh induces chondrogenesis and cartilage maturation in E12.5 limb micromass cultures, the effects of exogenous IHH protein and 5E1 were evaluated. Exogenous application of the IHH protein (500 ng/ml) resulted in an increase in the number of cartilage nodules and in the intensity of the alcian blue staining, whereas the addition of 5E1 caused a decrease in the number of cartilage nodules. Coincident to this, cartilage formation was increased or decreased following addition of the IHH protein or 5E1, respectively, in the optical density analysis (Fig. 4 A and B). This result indicates that Ihh induces early chondrogenesis consist of mesenchymal cell condensation, proliferation and differentiation into chondrocytes.

### Ihh Regulates *Runx2* and *Runx3* during Chondrogenesis

The addition of exogenous beads soaked in IHH protein increased *Gli1, Runx2* and *Runx3* expression in the mesenchymal cell pellets from the E12.5 limb buds at three and 24 hours after treatment, as shown by both *in situ* hybridizations and RT-qPCR (Fig. 4 C and D).

**Figure 5:**
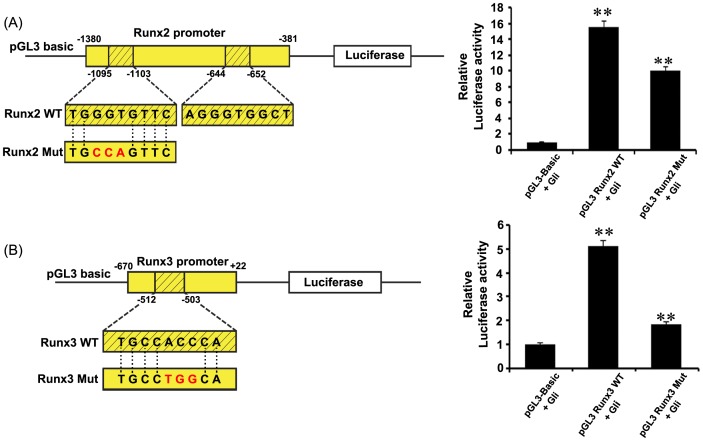
The Ihh-Gli pathway positively induces Runx2 and Runx3. (A) Compared to the pGL3-basic, treatment with 10 ng of the Gli1 expression vector leads to a significant increase of wild-type Runx2 containing Gli-binding site (pGL3-Runx2 WT, 500 ng), but the luciferase activity of Runx2 containing mutated Gli-binding site (pGL2-Runx2 Mut, 500 ng) is decreased, compared to pGL3-Runx2 WT. (B) pGL3-Runx3 WT luciferase activity increases significantly with the Gli expression vector, but pGL3-Runx3 Mut luciferase activity decreases than pGL3-Runx3 WT. Student’s t-test was employed for statistical analysis, with the level of statistical significance set at (**) *p*<0.005.

To determine whether Ihh up-regulates Runx2 and Runx3 expression directly, 293T cells were cotransfected with pFLAG-CMV3:Gli1 expression vector and either wild-type Runx2 containing Gli-binding site, or Runx2 mutants containing the mutation in their Gli-binding domain. Furthermore, pFLAG-CMV:Gli1 expression vector was cotransfected with either wild-type Runx3 or Runx3 mutants into 293T cells. As results, the relative luciferase activity of wild-type pGL3-Runx2 (Fig. 5 A) and wild-type pGL3-Runx3 (Fig. 5 B) was increased significantly with Gli1 expression constructs (10 ng), compared to the pGL3-basic. On the other hand, the relative luciferase activity of mutant-type pGL3-Runx2 (Fig. 5 A) and mutant-type pGL3-Runx3 (Fig. 5 B) was decreased than pGL3-Runx2 WT and pGL3-Runx3 WT. Therefore, this finding provides direct evidence showing that the Ihh-Gli pathway enhances the direct induction of both Runx2 and Runx3 transcription.

## Discussion

### Ihh is Required for Early Chondrogenesis in Developing Limbs

Two kinds of hedgehog are expressed in developing mouse limbs. *Shh* expression appeared in a zone of polarizing activity in mice prior to the onset of *Ihh* expression from E9.5 to E10.5. *Ihh* is expressed in condensed mesenchymal cells from E12.0, in prechondrocytes at the center of the limb cartilage at E12.5, and in prehypertrophic and hypertrophic chondrocytes in the developing growth plate from E14.5 [5]. *Shh^−/−^* mice show a normal stylopod phenotype, but their reduced or fused zeugopods and autopods show a partial loss of digits [28], [29]. *Ihh^−/−^* mice display reduced and partly fused stylopods, zeugopods and autopods [8]. Indeed, Shh is important in controlling digit number and patterning along the anterior-posterior axis of limb buds [30], [31], and *Ihh* is a regulator of endochondral bone formation [6].

In this study, we utilized the maternal transfer of 5E1 that leads to an attenuation of hedgehog activity in mouse embryos [19]. The binding efficiency of 5E1 to the Shh, Ihh and Dhh proteins was evaluated in a previous study; it was found that 5E1 (10 µg/ml) completely blocked the Shh (2 µg/ml)-induced differentiation of C3H10T1/2 cells, and its Ihh-binding activity is the same as its Shh-binding activity [18]. In this study, to rule out the function of Shh and focus on the function of Ihh in early chondrogenesis in limbs, 5E1 was transferred to mouse embryos at E12.5, E14.5, E16.5 and E18.5 because *Shh* is not expressed in the limb buds after E11.5 and *Dhh* is not expressed in limb buds at all.

The limbs of 5E1-treated mice did not exhibit the reduced number of phalanges observed in Shh^−/−^ mice. This difference indicates that injected 5E1 did not change the pattern of phalanges, which may be already determined prior to E12.5. However, the lengths of cartilage and bones were decreased in mice exposed to 5E1 at E12.5 only. Maternal transfer of 5E1 to E14.5, E16.5 and E18.5 embryos did not result in any morphological changes in their limbs, indicating that the pivotal event may occur from E12.5 to E14; this period is when mesenchymal cell condensation, proliferation, and differentiation into chondrocytes occur to form the cartilage primordia. Micromass cultures of limb bud mesenchymal cells have been well established as a model to analyze cell condensation, proliferation and differentiation in chondrogenesis [32]. In the present study, exogenous IHH protein caused mesenchymal cells to produce more cartilage nodules compared to PBS treatment, whereas 5E1 disrupted nodule formation. Therefore, Ihh may play an important role in condensation, proliferation and differentiation into chondrocytes.

**Figure 6:**
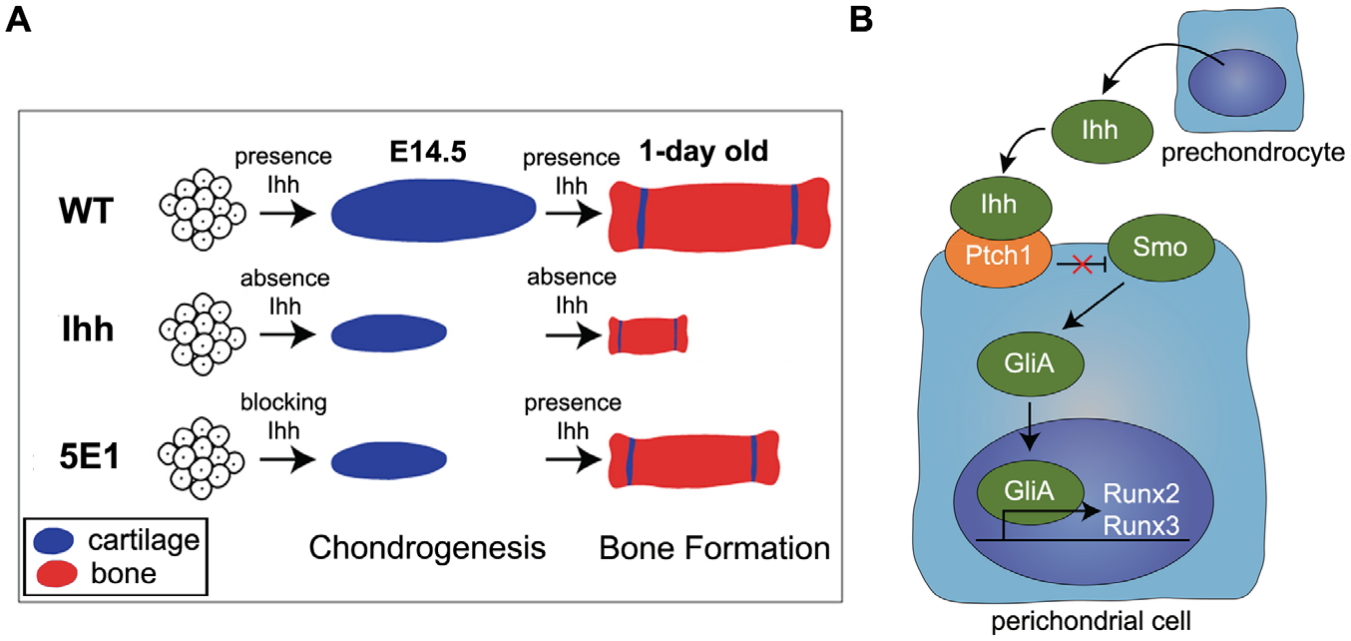
The differentiation of perichondrial cells was induced by Runx2 and Runx3 *via* the Ihh-Gli pathway. (A) The cartilage in the *Ihh ^−/−^* mutant limbs is shorter than that in the wild-type limbs at E14.5. This phenotype becomes progressively more severe by the time of birth, and the mice die at birth. When Ihh is blocked temporarily at E12.5, the cartilage primordium is shorter and similar to the Ihh^−/−^ mouse limbs at E14.5. Then, because Ihh is restored to the epiphyseal plate, the bone is well formed but short. Nevertheless, these short-boned mice can survive. (B) The binding of Ihh, which is the signal from prechondrocyte to the Ptch1 receptor of perichondrial cells, relieves Smo inhibition, leading to the activation of Gli. GliA then translocates to the nucleus to control the transcription of the Ihh target genes Runx2 and Runx3 to promote perichondrocyte differentiation.

Two days after injection at E12.5, the size of the cartilage primordia in stylopods, zeugopods and autopods was smaller in the 5E1-treated limbs compared to PBS-treated limbs. The length of each kind of bone was shorter in the 5E1-treated limbs, even in adults. However, each kind of cartilage and bone in the 5E1-treated limbs was longer than those in the *Ihh^−/−^* mice at birth, when most of the *Ihh* ^−/−^ mice died [8]. This difference may result from the difference in the duration of the blocking of Ihh activity between the 5E1-injected mice and the *Ihh^−/−^* mice. The treatment with 5E1 can block Ihh signaling activity temporarily, whereas *Ihh^−/−^* mice lose Ihh activity permanently (Fig. 6 A).

### Target Genes of Ihh are Down-regulated in the Putative Perichondrium

*Ihh^−/−^* mice show fewer perichondrial cells in the limb buds at E14.5 compared to wild-type mice [33]. *Ptch1, Hhip* and *Gli1*, direct targets of hedgehog signaling, are initially expressed at some distance from the edge of cartilage condensation; over time, the expression domain becomes restricted to the perichondrium proper [34], [35]. The *Ihh* ^−/−^ and *Smo* ^−/−^ mice show the reduced expression of *Ptch1* and *Gli1* in the perichondrium of E14 limbs [10]. Coincident to this, *Ptch1, Hhip* and *Gli1* expression in the putative perichondrium were down-regulated following 5E1 injection at E12.5. Furthermore, the *Gli1* expression level in limb mesenchymal cells was increased by exogenous IHH protein. Therefore, Ihh signaling may cause limb mesenchymal cells to form a perichondrium in early chondrogenesis.

### Ihh Positively Regulates Runx2 and Runx3 in the Putative Perichondrium

Runx2 and Runx3 are associated with the differentiation and maturation of chondrocytes and with osteoblast differentiation, as they are expressed in the perichondrium during early chondrogenesis. *Runx2* ^−/−^; *Runx3* ^−/−^ mice show shorter limbs than wild-type mice due to reduced and disorganized chondrocyte proliferation [12], [13], [16]. Runx2 plays a role in the length of stylopods, and Runx3 plays a role in the length of zeugopods and autopods [16], [17]. *Runx2* expression in limb chondrogenesis was shown to be regulated by Shh [15]. Furthermore, *Ihh* ^−/−^ mice were shown to lose the expression of *Runx2* in the perichondrial region at E13.5 [8]. In this study, the expression of both *Runx2* and *Runx3* in the perichondrium disappeared at one day after the blocking of Ihh activity. The expression levels of *Runx2* and *Runx3* were significantly increased by treatment with exogenous IHH protein. These results indicate that both Runx2 and Runx3 in the limb perichondrium are positively regulated by Ihh. Runx2 has already been reported as a target of hedgehog pathway using a luciferase reporter assay [15]. In this study, Gli1 expression constructs significantly increased the Runx2 promoter activity and Runx3 promoter activity in the luciferase reporter assay, providing evidence that the Ihh-Gli pathway directly enhances Runx2 and Runx3 transcription. Taken together, these findings suggested that Ihh binds to the Ptch1 receptor to relieve the inhibition of Smo, resulting in the translocation of Gli activator to the nucleus to regulate Runx2 and Runx3 (Fig. 6 B).

*Runx2* ^−/−^ mice delay chondrocyte hypertrophy in the stylopods rather than in other skeletal elements [17]. *Runx2* ^−/−^;*Runx3* ^+/−^ mice show short bone lengths in stylopods, while *Runx2* ^+/−^;*Runx3* ^−/−^ mice show short bones in zeugopods and autopods. *Runx2* ^−/−^;*Runx3* ^−/−^ mice display short limbs in stylopods, zeugopods and autopods together [16]. These studies indicate that Runx2 plays an important role in the bone length of stylopods and Runx3 in the bone length of zeugopods and autopods [16], [17]. The *Ihh* ^−/−^ mice and the 5E1-treated mice displayed short bones in their stylopods, zeugopods, and autopods, suggesting that not only Runx2 but also Runx3 are downstream genes of Ihh signaling in early chondrogenesis. On the other hand, it has been suggested that Runx2 coordinates chondrocyte proliferation and maturation by directly regulating *Ihh* expression, and Runx3 indirectly regulates *Ihh* expression [16]. This result suggests the possibility that early chondrogenesis is regulated by a feedback loop, in which Ihh in the center of the cartilage induces *Runx2* and *Runx3* expression in the perichondrium, followed by Runx2 and Runx3 induces *Ihh* expression in proliferating chondrocytes.

In summary, based on results showing the morphological and genetic changes resulting from the inhibition of Ihh signaling activity by 5E1 injection at E12.5, it is suggested that Ihh plays an important role in the formation of the cartilage anlagen and the perichondrium in limbs via harmonious and dynamic interactions with Runx2 and Runx3 during early chondrogenesis.

## Supporting Information

Figure S1 **Microarray.** In the microarray, the selected genes in limb showed a difference in regulation between the groups injected with either PBS or 5E1 at E12.5. The genes were selected differentially expressed genes in Wnt, TGF- β, Hedgehog, and Fgf signaling pathways with changes in expression of at least 1.5-fold in the 5E1-treated group compared to the control group. In addition, the hedgehog downstream genes, and the chondrogenesis- and osteogenesis-related genes were selected.
(TIF)

Figure S2 **Western blot.** For the western blot, embryos exposed to 5E1 or PBS were harvested two day after injection at E12.5. The translational level of Runx2 and Runx3 are reduced in 5E1-treated limb buds.
(TIF)
