# Supplementary figures and images for: Ihh and Runx2/Runx3 Signaling Interact to Coordinate Early Chondrogenesis: A Mouse Model
Source: PLoS One. 2013 Feb 1;8(2):e55296. doi: 10.1371/journal.pone.0055296 (PMC3562241; doi:10.1371/journal.pone.0055296)

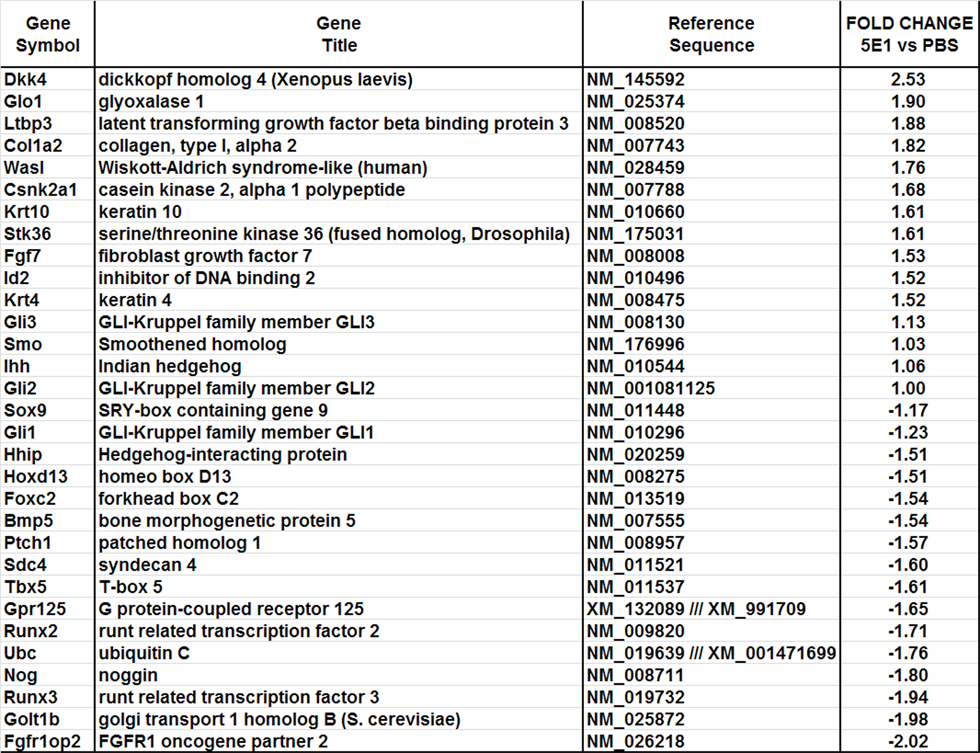

Supplement: Figure S1 — Microarray. In the microarray, the selected genes in limb showed a difference in regulation between the groups injected with either PBS or 5E1 at E12.5. The genes were selected differentially expressed genes in Wnt, TGF- β, Hedgehog, and Fgf signaling pathways with changes in expression of at least 1.5-fold in the 5E1-treated group compared to the control group. In addition, the hedgehog downstream genes, and the chondrogenesis- and osteogenesis-related genes were selected. (TIF) [file pone.0055296.s001.tif]

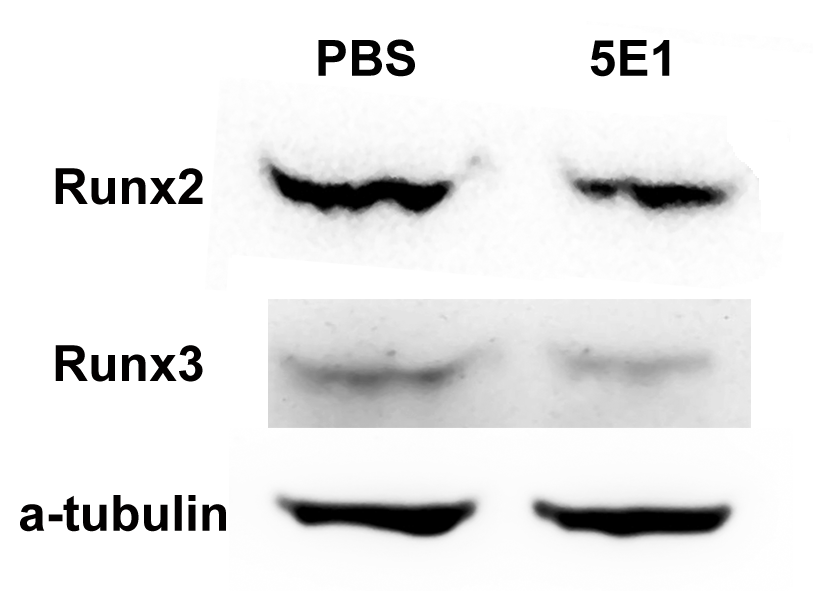

Supplement: Figure S2 — Western blot. For the western blot, embryos exposed to 5E1 or PBS were harvested two day after injection at E12.5. The translational level of Runx2 and Runx3 are reduced in 5E1-treated limb buds. (TIF) [file pone.0055296.s002.tif]
